# Integrated proteomics and metabolomics reveal mechanisms of blood pressure reduction in spontaneously hypertensive rats under hypoxic conditions

**DOI:** 10.3389/fphys.2026.1859072

**Published:** 2026-07-20

**Authors:** Delong Duo, Yan Wang, Guiqin Liu, Junbo Zhu, Yafeng Wang, Shanlu Bao, Xiangyang Li

**Affiliations:** 1 Qinghai Provincial People’s Hospital, Xining, China; 2 School of Pharmacy, Qinghai University, Xining, China; 3 Qinghai University Affiliated Hospital, Xining, China; 4 State Key Laboratory of Plateau Ecology and Agriculture, Qinghai University, Xining, China

**Keywords:** blood pressure, high-altitude hypoxia, metabolomics, proteomics, spontaneously hypertensive rats

## Abstract

**Introduction:**

High-altitude hypoxic environments markedly affect the cardiovascular system and blood pressure regulation. However, the molecular mechanisms underlying decreases in blood pressure induced by chronic hypoxia exposure in spontaneously hypertensive rats (SHRs) remain unclear. Therefore, we systematically elucidated the mechanisms by which chronic high-altitude hypoxia exposure induces adaptive molecular remodeling and subsequent decreases in blood pressure SHRs.

**Methods:**

SHRs were randomly divided into control (SHR-C, 1660 m; PaO_2_, 17.5 kPa, 10 weeks) and high-altitude hypoxia (SHR-H; 4300 m; PaO_2_, 12.5 kPa, 10 weeks) groups. Tail artery blood pressure was monitored, and abdominal aortic tissues underwent tandem mass tag-labeled quantitative proteomics and untargeted metabolomics analyses. Bioinformatics analyses were conducted for molecular screening of differential expression, functional enrichment, and network integration, followed by key protein validation via western blotting.

**Results and discussion:**

The SHR-H group exhibited pronounced reductions in systolic blood pressure, diastolic blood pressure, and mean arterial pressure compared with the SHR-C group. Proteomics analysis identified 185 differentially expressed proteins (161 upregulated and 24 downregulated). According to functional enrichment analysis, the upregulated proteins were considerably enriched in energy metabolism pathways, whereas downregulated proteins were associated with inflammatory and stress responses. Integrated protein–metabolite network analysis revealed that the tricarboxylic acid cycle was the central hub, and western blotting validated the upregulation of mitochondrial-associated proteins. Metabolomics confirmed energy metabolism reprogramming by detecting markers of enhanced fatty acid oxidation. Overall, this study provides correlative mechanistic insights into the cardiovascular effects of high-altitude hypoxic environments and proposes novel metabolic intervention strategies for hypertension, though direct functional validation is necessary to confirm the proposed mechanisms.

## Graphical Abstract

**Figure:**
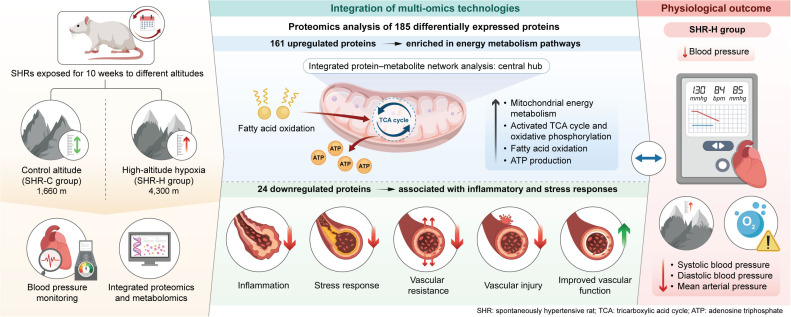

## 1 Introduction

Areas >2,500 m above sea level are defined as being high-altitude. Over 81.6 million people live in high-altitude areas; of them, 14.4 million reside permanently at altitudes >3,500 m. The populaces of the Andean mountains of South America, the Himalayan mountains of Tibet, and the Ethiopian summits of Africa have the longest histories of high-altitude residency (Tremblay and Ainslie, 2021). Such high-altitude environments are characterized by intense ultraviolet radiation, low humidity, diurnal temperature variations, poor soil quality, and hypobaric hypoxia, which are the primary factors affecting human activity (Zhu et al., 2022). Additionally, high-altitude hypoxic environments markedly affect the cardiovascular system and blood pressure regulation (Narvaez-Guerra et al., 2018).

The number of adults with hypertension worldwide is predicted to reach 1.56 billion by 2025 (Kearney et al., 2005). Hypertension is a major risk factor for cardiovascular disease mortality and causes over seven million deaths annually worldwide (Danaei et al., 2011). Vascular metabolic dysfunction is crucial in the onset and progression of hypertension (Gallo et al., 2021). Specifically, mitochondrial dysfunction in vascular smooth muscle cells, accompanied by oxidative stress and chronic inflammation, leads to vascular remodeling, endothelial dysfunction, and increased peripheral resistance (Qin et al., 2023).

The impact of high-altitude hypoxic environments on the cardiovascular system is thus complex and multifaceted. Such environments can induce or exacerbate hypertension in susceptible individuals; however, under specific conditions, adaptive reductions in blood pressure have been observed. This adaptive change may be related to genetic, molecular, and physiological mechanisms; thus, further research to elucidate the precise roles of these mechanisms is necessary (Cornwell et al., 2021; Lyamina et al., 2011). Chronic high-altitude hypoxia exposure markedly decreases blood pressure in spontaneously hypertensive rats (SHRs); however, the underlying molecular mechanisms have not been systematically elucidated (Duo et al., 2025).

Integrated multi-omics approaches offer powerful tools for elucidating such complex mechanisms (Ohashi et al., 2015). In particular, tandem mass tags (TMT) offer a differential proteomics techniques for comprehensively and quantitatively describing protein expression and its changes under biological perturbations, such as diseases or drug treatments (Delles et al., 2012). Untargeted metabolomics involves a comprehensive metabolomic analysis of the entire organism, which provides profound information regarding the metabolite composition, metabolic pathways, and metabolic networks of the organism (Zhang et al., 2023). The combination of these approaches has recently attracted increasing attention.

The aim of this study was to elucidate the molecular mechanisms underlying decreases in blood pressure in SHRs under a high-altitude hypoxic environment by integrating TMT-based proteomics and untargeted metabolomics analyses of abdominal aortic tissues.

## 2 Methods

### 2.1 Reagents

(2-[4-(2-hydroxyethyl)piperazin-1-yl]ethanesulfonic acid, dithiothreitol, iodoacetamide, sodium deoxycholate (SDC), ammonium bicarbonate, anhydrous acetonitrile, hydroxylamine, formic acid, a protease inhibitor cocktail, methanol, acetonitrile, and ammonium acetate were all purchased from Sigma-Aldrich (St. Louis, MO, USA). Trypsin was purchased from Promega (Madison, WI, USA). Nonidet P40 (NP-40) and sodium dodecyl sulfate (SDS) were purchased from Sangon Biotechnology (Shanghai, China). The TMT Isobaric Label Reagent Set (6-plex, 10-plex, and 16-plex) was purchased from Thermo Fisher Scientific (Waltham, MA, USA). An enhanced bicinchoninic acid (BCA) protein assay kit was purchased from Beyotime (Shanghai, China). Anti-β-actin, cytochrome c oxidase subunit 5A (Cox5a), kininogen 1 (Kng1), and NADH:ubiquinone oxidoreductase core subunit S1 (Ndufs1) antibodies were obtained from Abclonal (Wuhan, China), whereas anti-acyl-coenzyme (CoA) synthetase long-chain family member 1 (AcsI1) and carnitine palmitoyltransferase 2 (Cpt2) antibodies were obtained from Huabio (Hangzhou, China). Anti-ubiquinol-cytochrome c reductase core protein 2 (Uqcrc2) was obtained from Proteintech (Chicago, IL, USA).

### 2.2 Animals and experimental treatments

Experiments were conducted on eight-week-old male SHRs purchased from Beijing Huafukang Biotechnology Co., Ltd. (Beijing, China) (certificate No: 2019-0008). The rats were housed in an animal facility on a 12-h light/dark cycle under a constant temperature of 22 ± 2 °C and humidity of 55 ± 10%, with food and water provided *ad libitum*. All animal experiments were conducted following the Regulations for the Administration of Affairs Concerning Experimental Animals (2017 revision) and the Laboratory Animals: General Requirements for Animal Experiments (GB/T 35823-2018). All experiments were approved by the Ethics Committee of Experimental Animal Use of Qinghai University (approval number: PJ202401-74; 15 March 2024).

The rats were exposed to high-altitude or control conditions for ten weeks and then studied, with six rats in each group: high-altitude hypoxia (SHR-H; 4300 m; partial pressure of oxygen in arterial blood [PaO_2_], 12.5 kPa) and control (SHR-C, 1660 m; PaO_2_, 17.5 kPa). Rats in the SHR-C group were maintained at an altitude of approximately 1,660 m in Minhe County, Qinghai Province, China, whereas those in the SHR-H group were maintained at an altitude of 4,000 m in Hua Shixia Town, Qinghai Province, China.

### 2.3 Blood pressure measurements

The systolic blood pressure (SBP), diastolic blood pressure (DBP), mean blood pressure (MBP), and heart rate (HR) at rest were measured using the tail-cuff method with a BP-2010 automatic noninvasive blood pressure monitor (Softron Biotechnology Co., Ltd, Beijing, China) (Liu et al., 2023a). Prior to resting-state the measurements, the rats were acclimated to the procedure. During the measurement, each rat was secured in a mesh bag, placed in a warming sleeve, and placed in a rat holder. Finally, the pressure sensor was aligned with the base of the tail. Three consecutive measurements were obtained, and mean values were calculated. After the final measurement, the rats were anesthetized, and the abdominal aorta was rapidly isolated and stored at −80 °C.

### 2.4 TMT proteomic assay

#### 2.4.1 Sample preparation

The samples were mixed thoroughly with radioimmunoprecipitation lysis buffer, and steel beads were added for low-temperature grinding. The samples were added to a cell disruptor placed in an ice-water bath for sonication to allow the samples to dissolve completely. Centrifugation was conducted at 12,000 ×*g* and 4 °C for 10 min, and the protein concentrations of the samples were quantified using a BCA assay kit.

#### 2.4.2 Digestion and TMT labeling

For protein purification, acetone was added to the samples, and the mixture was precipitated overnight at −20 °C and centrifuged. The pellet was resuspended in lysis buffer supplemented with 1% (w/v) SDC to improve protein solubilization and enzymatic digestion efficiency. Subsequently, proteins were reduced using 5 mM dithiothreitol at 55°C for 20 min, followed by alkylation with 15 mM iodoacetamide at room temperature for 30 min in the dark. For digestion, trypsin was added at a 1:50 (w/w) enzyme-to-protein ratio and incubated at 37 °C overnight. The samples were labeled using the Thermo TMT labeling kit according to the manufacturer’s instructions. Trifluoroacetic acid was added to the mixed sample, and the sample was mixed well to precipitate the SDC. The raw reporter ion intensities were median-normalized using SpectroMine software to correct for channel-to channel variation. The co-precipitated peptides were extracted to obtain the labeled peptide samples. The supernatant was collected for mass spectrometry (MS) using a C18 desalting-spinning column.

### 2.5 High-pH pre-fractionation

After lyophilization, the peptide samples were reconstituted in 50 μL of mobile phase A and separated under alkaline conditions using RPUPLC at a flow rate of 0.3 mL/min with a 150 × 2.1 mm Waters, XBridge BEH C18 XP column. Mobile phase A comprised a 10 mM aqueous ammonium acetate solution at pH 10, and mobile phase B comprised 10 mM ammonium acetate, 10% H_2_O, and 90% acetonitrile at pH 10. The liquid phase gradient was 60 min: 5% mobile phase B for 2 min, 5–30% for 40 min, 30–40% for 10 min, 40–90% for 4 min, and 90% for 2 min. One fraction was collected per minute, combined into twelve fractions, vacuum-dried, and stored at −80 °C.

### 2.6 Nano liquid chromatography-tandem MS analysis

For each sample, 2 μL of the total peptide solution was injected into a nano-ultra-performance liquid chromatography (UPLC) system (EASY-nLC1200) connected to an Orbitrap Exploris 480 mass spectrometer (Thermo Fisher Scientific) with a nano-electrospray ionization source. Separation was performed using a reversed-phase column (100 *µ*m ID ×15 cm, Reprosil-Pur 120 C18-AQ, 1.9 *µ*m, Dr. Maisch). The separation solvents included water with 0.1% formic acid and 2% acetonitrile (Phase A) and 80% acetonitrile with 0.1% formic acid (Phase B). A 90-min gradient at a flow rate of 300 µL/min facilitated peptide elution, as follows: 2% to 5% B over 2 min, 5% to 22% B over 68 min, 22% to 45% B over 16 min, increased to 95% B in 2 min, and held for 2 min. Data-dependent acquisition was performed in profile and positive modes. MS1 scans were obtained at a resolution of 120,000 at 200 m/z, scanning from 350 to 1600 m/z. MS2 scans were set at a resolution of 45,000 with a fixed start at m/z 110. The automatic gain control targets were set at 3E6, with a maximum injection time of 30 ms for MS1 and 1E5, and a maximum injection time of 96 ms for MS2. The top 20 most intense ions were fragmented using high-energy collisional dissociation with a normalized collision energy (NCE) of 32% and an isolation window of 0.7 m/z. A dynamic exclusion period of 45 s was applied, and ions with a single charge or more than six charges from selection were excluded.

### 2.7 Untargeted metabolomics assay

Abdominal aortic tissue samples from SHRs were collected, mixed with beads, and 500 μL of the extraction solution (methanol:acetonitrile:water = 2:2:1, v/v/v) was added. The extraction solution contained a deuterated internal standard. The mixed solution was vortexed for 30 s, sonicated for 10 min in a water bath (4 °C), and incubated for 1 h at −40 °C. Subsequently, the samples were centrifuged at 12,000 × *g* for 15 min at 4 °C. The supernatant was then transferred to a fresh glass vial for further analysis. Liquid chromatography tandem MS (MS/MS) analyses were conducted using an ultra-HPLC system (Thermo Fisher Scientific) linked to an Orbitrap Exploris 120 mass spectrometer (Thermo Fisher Scientific). The system utilized a water ACQUITY UPLC BEH Amide column (2.1 × 50 mm, 1.7 μm). The mobile phase was composed of 25 mmol/L ammonium acetate and 25 mmol/L ammonia hydroxide in water for Phase A, and acetonitrile was used as Phase B. The autosampler was maintained at 4 °C, with the injection volume set at 2 μL. MS/MS spectra were acquired using an Orbitrap Exploris 120 mass spectrometer operating in the information-dependent acquisition mode, managed by acquisition software that continuously evaluated the full-scan MS spectrum. For heatmap visualization, metabolite intensities were Z-score normalized across rows. The electrospray ionization source conditions were established with a sheath gas flow rate of 50 Arb, auxiliary gas flow rate of 15 Arb, capillary temperature of 320 °C, full MS resolution of 60,000, MS/MS resolution of 15,000, collision energy settings of stepped NCE 20/30/40, and spray voltage set at 3.8 kV for the positive mode or −3.4 kV for the negative mode.

#### 2.7.1 Functional categorization and pathway mapping

The raw protein MS files were processed using SpectroMine software and the built-in Pulsar search engine. The raw metabolite data were converted to mzXML format using ProteoWizard and processed using an in-house program developed using R and based on XCMS for peak detection, extraction, alignment, and integration. The R package and BiotreeDB were used for metabolite identification. Principal component analysis (PCA) and orthogonal projections to latent discriminant analysis (OPLS-DA) were among the multivariate statistical methods utilized.

Hierarchical clustering was performed using the heatmap and ggplot2 functions available in the R package, with results presented via heatmaps and volcano plots. The volcano plot was generated based on all detected metabolite features prior to MS2 based identification, including both identified and unidentified features. Annotation analysis of differentially expressed proteins (DEPs) was performed using the Gene Ontology (GO) and Kyoto Encyclopedia of Genes and Genomes (KEGG) databases (Kanehisa et al., 2016) (https://www.genome.jp/kegg/). The protein–protein interaction (PPI) networks were constructed, and key node proteins were identified using the STRING database (https://cn.string-db.org) and Cytoscape software (Szklarczyk et al., 2019).

### 2.8 Western blotting

Total protein was extracted from rat abdominal aortic tissue, and the protein concentration was determined using the BCA assay. Protein samples were separated by electrophoresis on 10% SDS polyacrylamide gel and transferred onto methanol-activated polyvinylidene fluoride membranes. After transfer, membranes were blocked with 5% non-fat milk at room temperature for 2 h, followed by 12–16 h of incubation at 4 °C with primary antibodies against β-actin (1:50000, Abclonal, AC026), AcsI1 (1:2000, Huabio, ER60807), Cox5a (1:2000, Abclonal, A6437), Cpt2 (1:1000, Huabio, ET1611-64), Kng1 (1:1000, Abclonal, A1670), Ndufs1 (1:2000, Abclonal, A21192), and Uqcrc2 (1:2000, Proteinetch, 14742-1-AP). Subsequently, the membranes were incubated at 25 °C with secondary antibodies diluted 1:8000 for 120 min and washed three times with Tris-buffered saline with Tween 20. Finally, protein bands were visualized using enhanced chemiluminescent substrate. Images were captured using a chemiluminescence imaging system, and band integral optical density was analyzed using Gel-Pro Analyzer 4 software (Media Cybernetics, Rockville, MD, USA) to quantify target protein expression levels.

### 2.9 Data analysis

All statistical analyses were conducted using SPSS version 27.0 (IBM Corp., Armonk, NY, USA). All data are expressed as the mean ± standard deviation. The criteria for screening DEPs were as follows: P < 0.05 and fold change of ≤0.5 or ≥2; proteins were considered downregulated or upregulated if the fold change was ≤0.5 or ≥2, respectively. For proteomic data, P-values were adjusted using the Benjamini-Hochberg method for FDR correction, and the corrected P-values were used for differential expression screening. Volcano plots and hierarchical clustering heatmaps were generated using the R packages ggplot2 and pheatmap, respectively. Metabolomics data were analyzed using SIMCA software to log-transform and center the data. Subsequently, OPLS-DA automatic modeling analysis and Student’s t-test were conducted to obtain the variable importance in projection (VIP) and P-value. Differential metabolites were identified by screening for metabolites with VIP > 1 and P < 0.05. All samples were processed and analyzed in a single batch, thus, batch effect correction was not applicable.

Integration of proteomics and metabolomics datasets was performed using a multi-step strategy. First, pathway-level co-analysis was performed by identifying shared KEGG pathways from the enrichment results. PPI networks were constructed using the STRING database and visualized with Cytoscape software. Differential metabolites were then mapped to KEGG pathways to identify commonly enriched pathways. Second, a correlation network was constructed by performing Spearman correlation analysis between DEPs and differential metabolites using the cor.test function in R. Only correlations with a P < 0.05 were retained for network construction in Cytoscape. Third, network centrality analysis was conducted by calculating node centrality scores using the hub_score function in the igraph R package, and key hub nodes were subsequently identified and visualized with Cytoscape. Finally, all network graphs were generated and visualized using Cytoscape.

## 3 Results

### 3.1 Effects of high-altitude hypoxic conditions on SBP, DBP, MBP, and HR

**Figure 1:**
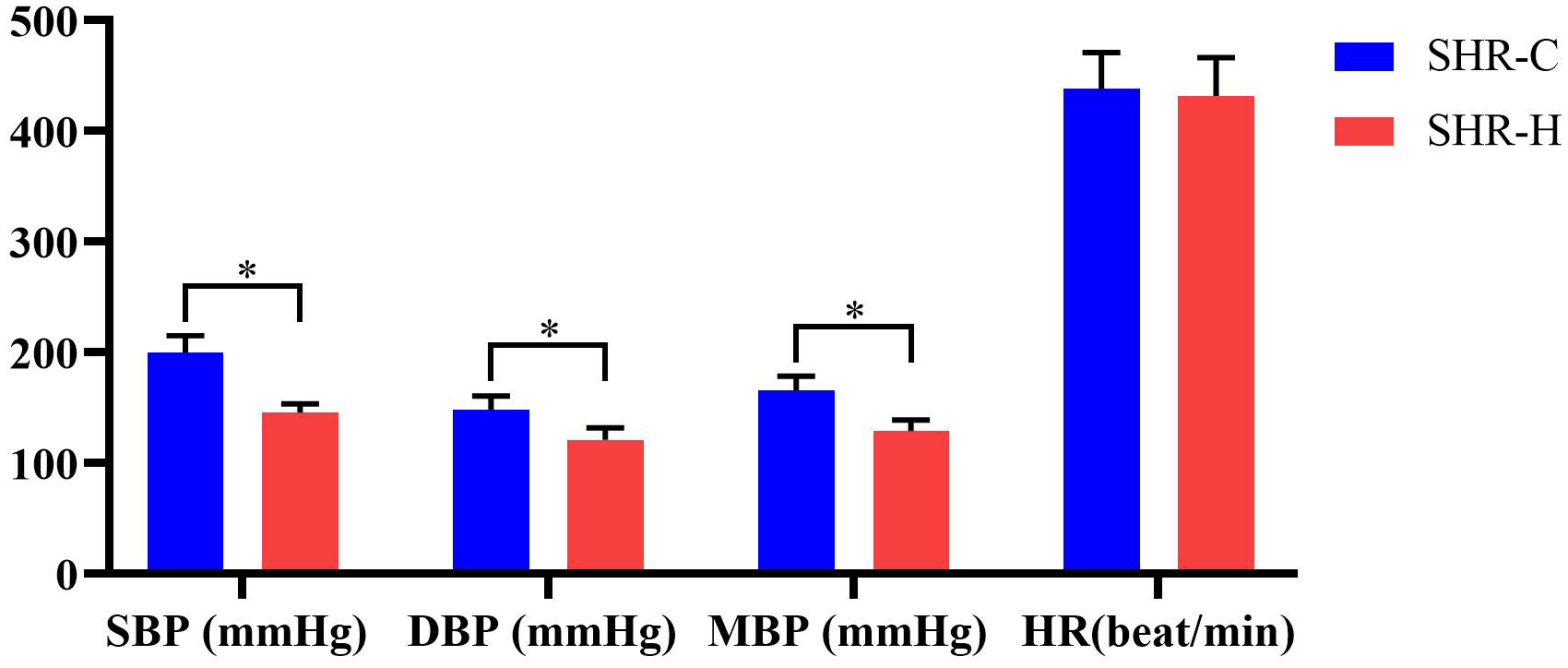
Changes in systolic blood pressure (SBP), diastolic blood pressure (DBP), mean arterial pressure (MBP), and heart rate (HR) in spontaneously hypertensive rats (SHR) after exposure to high-altitude hypoxic conditions. Data are presented as the mean ± SD (n = 6). Note: SHR-C and SHR-H refer to the SHR control group and SHR high-altitude hypoxia group, respectively. *P < 0.05 vs. SHR-C (*t*-test).

After 10 weeks of exposure to high-altitude hypoxic conditions, the SHR-H group showed significant decreases in SBP, DBP, and MBP compared with the SHR-C group (146 ± 8 vs. 200 ± 16 mmHg, 121 ± 11 vs. 148 ± 12 mmHg, and 129 ± 10 vs. 166 ± 13 mmHg, respectively; P < 0.05). No significant differences in HR were observed between the SHR-H and SHR-C groups (P > 0.05) (**Figure 1**).

### 3.2 TMT-based proteomic analysis

**Figure 2:**
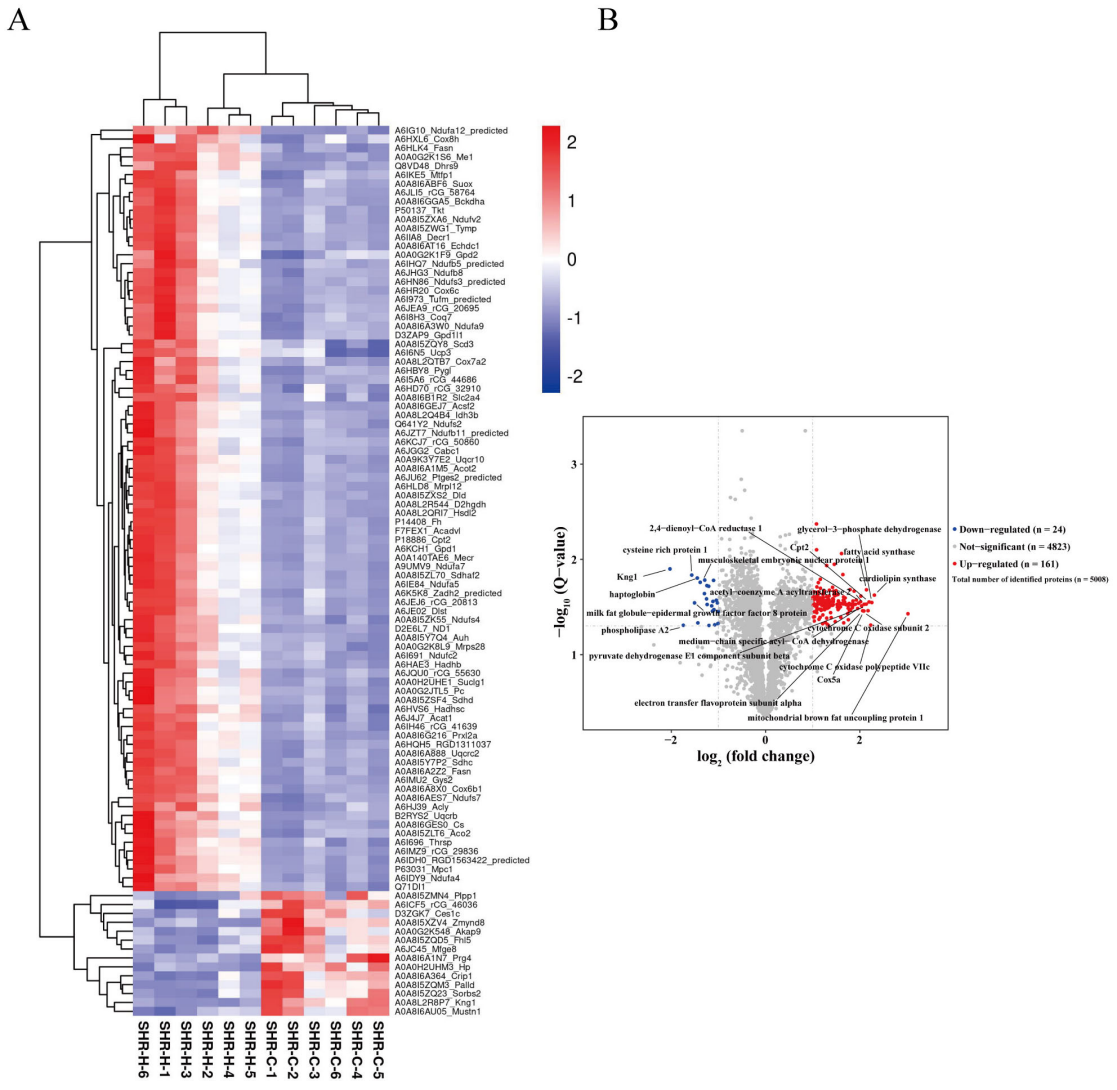
Changes in the proteomic profile of the abdominal aorta in spontaneously hypertensive rats (SHR) after exposure to high-altitude hypoxic conditions. **(A)** Hierarchical clustering heatmap of differentially expressed proteins (DEP) Protein intensities were Z-score normalized across rows (red: upregulation, blue: downregulation). **(B)** Volcano plot showing the distribution of all quantified proteins. Data were screened based on a fold change threshold of ≥ 2 or ≤ 0.5 and an adjusted *P* < 0.05 (n = 6). SHR-C and SHR-H refer to the SHR control group and SHR high-altitude hypoxia group, respectively.

After 10 weeks of exposure to high-altitude hypoxia, abdominal aortic tissue samples were collected from the SHRs. DEPs were screened using TMT-labeled proteomics. The criteria for screening DEPs were unique peptides (P < 0.05) and a fold change of ≤0.5 or ≥ 2. In total, 185 DEPs were identified, of which 161 were upregulated and 24 were downregulated (**Supplementary Table 1**). The heatmap of DEPs between the SHR-C and SHR-H groups showed that 10 weeks of exposure to high-altitude hypoxia exposure caused pronounced changes in protein levels in the abdominal aortic tissue samples from the SHR-H group compared with those from SHR-C group (**Figure 2A****).** Volcano plots revealed that mitochondrial brown fat uncoupling protein 1, cardiolipin synthase, glycerol-3-phosphate dehydrogenase, cytochrome C oxidase polypeptide VIIc, Cpt2, cytochrome C oxidase subunit 2, fatty acid synthase, pyruvate dehydrogenase E1 component subunit beta, acetyl-coenzyme A acyltransferase 2, Cox5a, electron transfer flavoprotein subunit alpha, medium-chain specific acyl- CoA dehydrogenase, RCG29836, and 2,4-dienoyl-CoA reductase were the most substantially upregulated proteins, with a cutoff of four-fold, whereas Kng1, phospholipase A2, cysteine rich protein 1, milk fat globule epidermal growth factor 8 protein, haptoglobin, RCG22430, and musculoskeletal embryonic nuclear protein 1 were markedly downregulated (**Figure 2B**).

### 3.3 Enrichment analysis of DEPs

**Figure 3:**
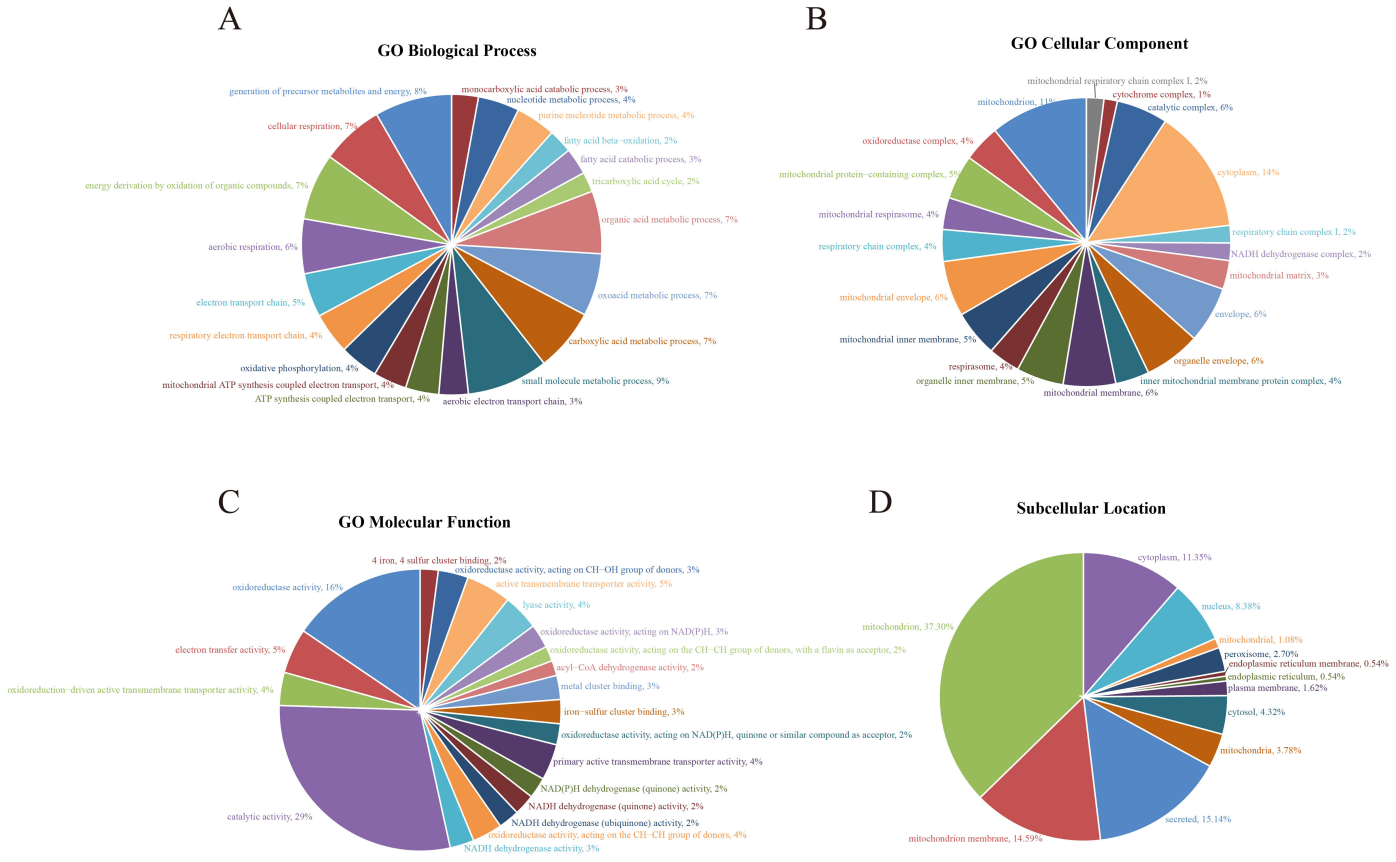
Changes in Gene Ontology (GO) functional annotation of differentially expressed proteins (DEPs) in the abdominal aorta of spontaneously hypertensive rats (SHR) after exposure to high-altitude hypoxic conditions. Enrichment analysis of DEPs in **(A)** Biological processes. **(B)** Cellular component. **(C)** Molecular function. **(D)** Subcellular location. Data are presented as the number of the DEPs associated with each GO term (n = 6). SHR-C and SHR-H refer to the SHR control group and SHR high-altitude hypoxia group, respectively.

GO analysis was conducted to comprehensively understand the biological significance of the 185 DEPs. This analysis technique explores the biological processes in which proteins participate, the cellular components in which they are located, and the molecular functions they perform (Dennis et al., 2003; Huang et al., 2009). Additionally, the subcellular localization of these proteins has been predicted (Yu et al., 2006). Our results indicated that most of the altered proteins mainly participated in the generation of precursor metabolites and energy, cellular respiration, energy derivation by oxidation of organic compound oxidation, organic acid metabolic processes, oxoacid metabolic processes, aerobic respiration, and the electron transport chain (**Figure 3A**). Furthermore, GO annotation demonstrated that the altered proteins were located in the cytoplasm, mitochondrion, catalytic complex, mitochondrial envelope, envelope, organelle envelope, mitochondrial membrane, mitochondrial protein-containing complex, mitochondrial inner membrane, organelle inner membrane, oxidoreductase complex, mitochondrial respirasome, respiratory chain complex, respirasome, and inner mitochondrial membrane protein complex (**Figure 3B**). Functional analyses demonstrated that many of the proteins were associated with catalytic, oxidoreductase, electron transfer, active transmembrane transporter, lyase, oxidoreduction-driven active transmembrane transporter, primary active transmembrane transporter, and oxidoreductase activity on the CH-OH group of donors (**Figure 3C**). The subcellular localizations of these DEPs were primarily the mitochondrion, secretions, mitochondrion membrane, cytoplasm, nucleus, cytosol, mitochondria, peroxisome, plasma membrane, and mitochondrial (**Figure 3D**).

**Figure 4:**
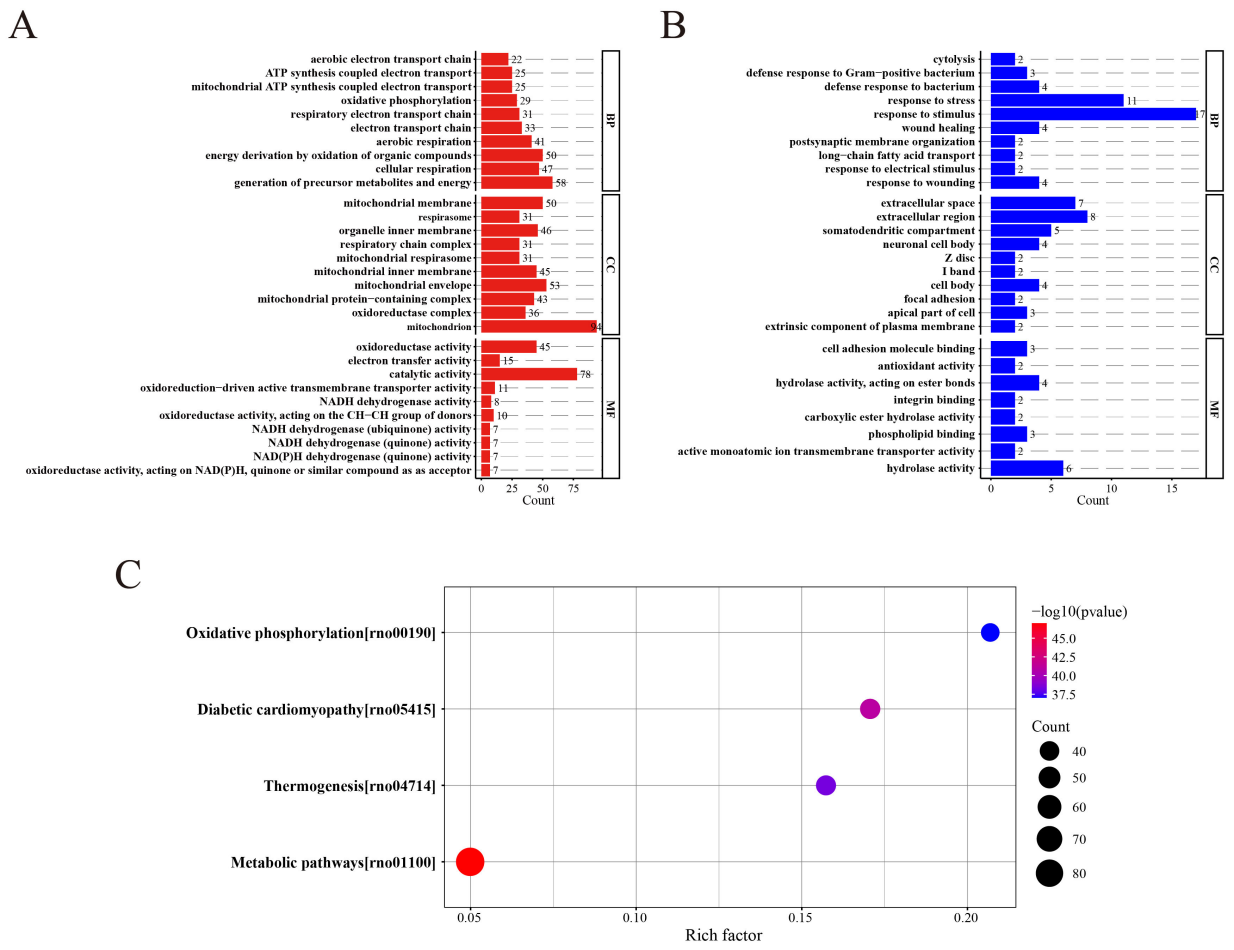
Changes in functional enrichment pathways of differentially expressed proteins (DEPs) in the abdominal aorta of spontaneously hypertensive rats (SHR) after exposure to high-altitude hypoxic conditions. **(A)** Gene ontology (GO) enrichment analysis of upregulated proteins. **(B)** GO enrichment analysis of downregulated proteins. **(C)** Kyoto Encyclopedia of Genes and Genomes (KEGG) pathway enrichment analysis. Data are presented as the -log10(*P*-value) or number of the DEPs associated with each GO term/pathway (n = 6). SHR-C and SHR-H refer to the SHR control group and SHR high-altitude hypoxia group, respectively.

To further elucidate the adaptive strategies under high-altitude hypoxia, GO enrichment analysis was independently performed on upregulated and downregulated proteins. This analysis revealed a bifurcated regulatory pattern, in which the upregulated proteins were overwhelmingly enriched in biological processes essential for energy metabolism, including the generation of precursor metabolites and energy, as well as energy derivation by the oxidation of organic compounds. Their cellular components were distinctly localized to the mitochondria and mitochondrial envelope, and their molecular function was centered on catalytic and oxidoreductase activities. In contrast, downregulated proteins were predominantly enriched in processes related to response to stimuli and stress. which were located in the extracellular region and space, and were functionally characterized by hydrolase activity and hydrolase activity acting on ester bonds (**Figures 4A, B**).

DEPs were subjected to KEGG pathway enrichment analysis in order to identify their biological processes and functions. The top four most substantially enriched pathways are shown in **Figure 4C**. Metabolic pathways were the most substantially enriched, followed by thermogenesis, diabetic cardiomyopathy, and oxidative phosphorylation.

### 3.4 Untargeted metabolomics

**Figure 5:**
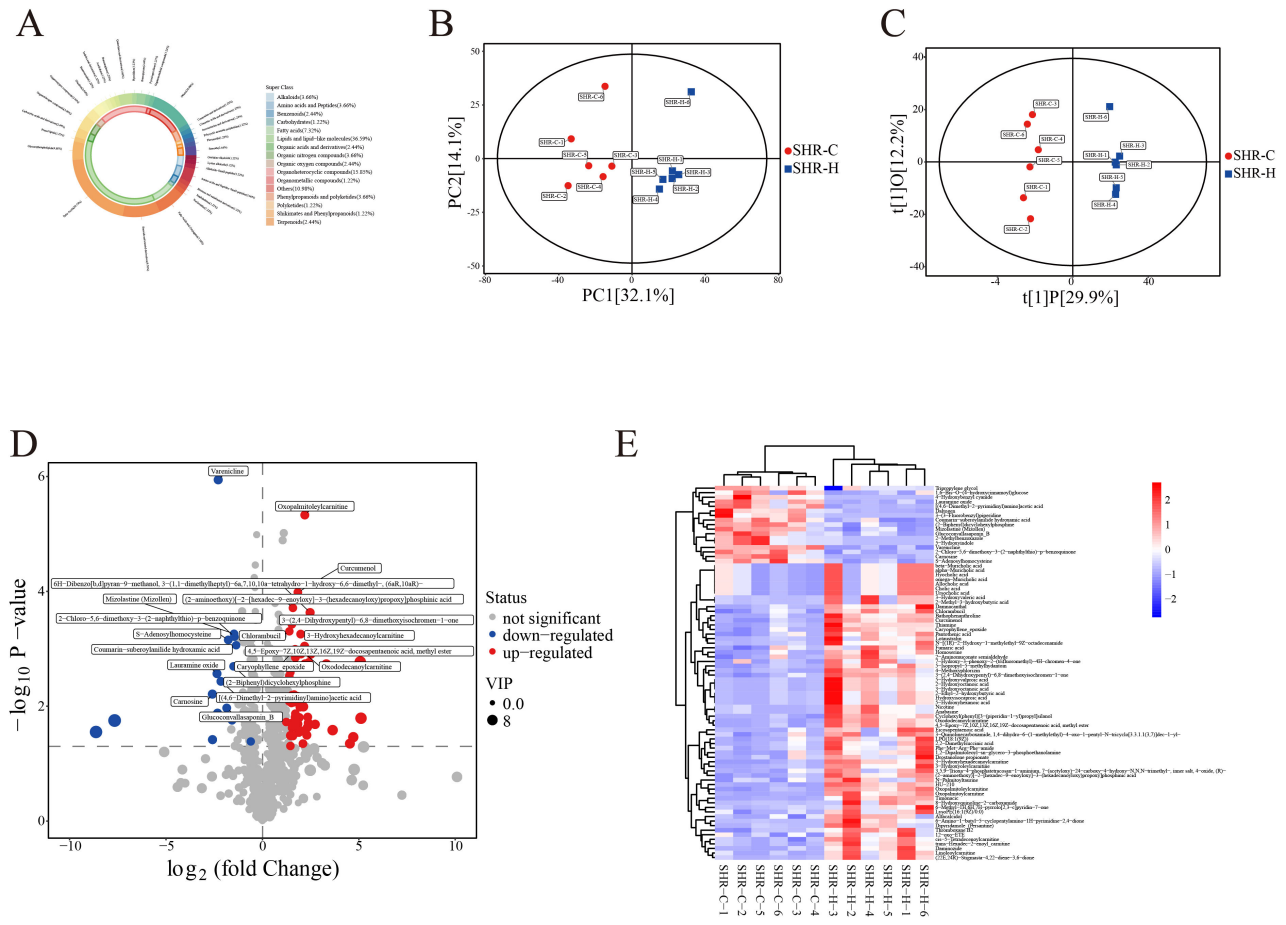
Changes in the metabolomic profile of the abdominal aorta in spontaneously hypertensive rats (SHR) after exposure to high-altitude hypoxic conditions. **(A)** Donut plot of metabolite classification and proportions. **(B)** Principal component analysis (PCA) score plot. **(C)** Orthogonal projections to latent structures-discriminant analysis (OPLS-DA) score plot (Z-score normalized across rows). **(D)** Volcano plots of all detected metabolite features. **(E)** Hierarchical clustering heatmap of differential metabolites. Differential metabolites were screened based on a variable importance in projection (VIP) > 1 and *P* < 0.05 (n = 6). Note: SHR-C and SHR-H refer to the SHR control group and SHR high-altitude hypoxia group, respectively.

A metabolomic analysis was conducted using UHPLC/MS. In total, 1,184 metabolites were identified and classified. Lipids and lipid-like molecules constituted the most abundant category (36.59%), followed by organoheterocyclic compounds (15.85%), highlighting a predominant shift in lipid metabolism under high-altitude hypoxic conditions (**Figure 5A**). Pattern recognition analysis was conducted via PCA and OPLS-DA to investigate the metabolites. These analyses revealed pronounced differences in metabolites between the SHR-H and SHR-C groups exposed to high-altitude hypoxia (**Figures 5B, C**). In order to identify potential metabolic biomarkers in rats exposed to high-altitude hypoxia, 1,184 differentially expressed peaks were identified in the SHR-H group (P < 0.05 and VIP ≥ 1). Among them, 82 differentially expressed metabolites, including 65 upregulated and 17 downregulated metabolites, were identified using secondary MS matching analysis. The full spectrum of detected metabolite features was visualized using volcano plots. Notably, several acylcarnitines, including oxopalmitoleylcarnitine, 3-hydroxyhexadecanoylcarnitine, and oxododecanoylcarnitine, which serve as direct biomarkers of enhanced mitochondrial fatty acid β-oxidation, were markedly upregulated. Other upregulated metabolites included curcumenol and HU-210. The significantly downregulated metabolites included varenicline, mizolastine (mizollen), 2-chloro-5,6-dimethoxy-3-(2-naphthylthio)-p-benzoquinone, S-adenosylhomocysteine, coumarin-suberoylanilide hydroxamic acid, (2-biphenyl)dicyclohexylphosphine, lauramine oxide, [(4,6-dimethyl-2-pyrimidinyl)amino]acetic acid, carnosine, and glucoconvallasaponin_B (**Figure 5D**; **Supplementary Table 2**). Hierarchical clustering analysis using a heatmap of the differentially expressed metabolites between the SHR-C and SHR-H groups showed that 10 weeks of high-altitude hypoxia exposure caused substantial changes in metabolite levels, characterized by a shift in energy metabolism towards the utilization of fatty acids and accompanied by a decreases in metabolites, such as S-adenosylhomocysteine and carnosine (**Figure 5E**).

### 3.5 Metabolic pathway enrichment analysis

**Figure 6:**
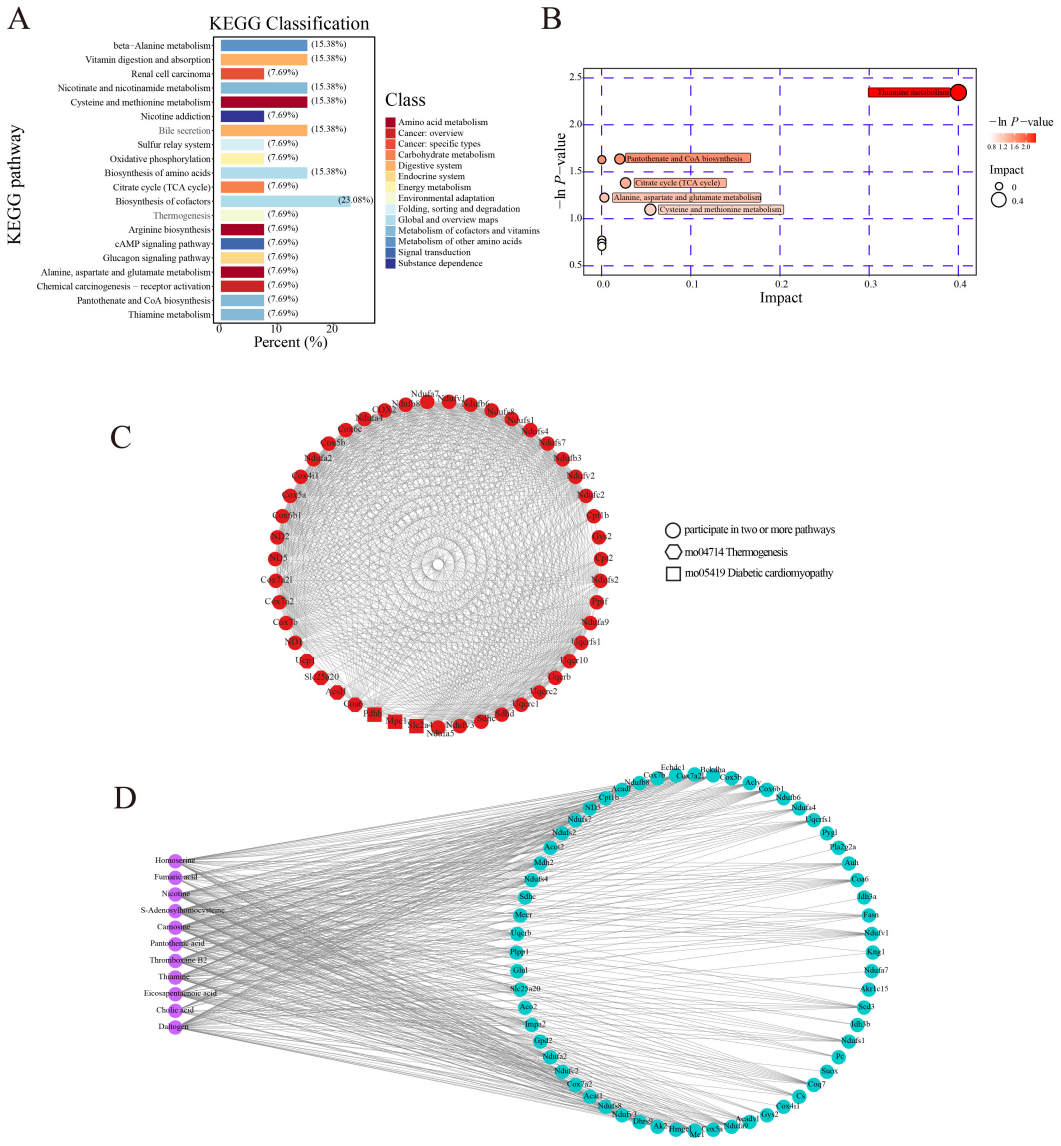
Changes in network and pathway integration of proteomics and metabolomics data in the abdominal aorta of spontaneously hypertensive rats (SHR) after exposure to high-altitude hypoxic conditions. **(A)** KEGG classification of differential metabolites. **(B)** Metabolic pathway enrichment analysis of differential metabolites. **(C)** PPI network of DEPs. **(D)** Integrated protein-metabolite interaction network. SHR-C and SHR-H refer to the SHR control group and SHR high-altitude hypoxia group, respectively.

KEGG pathway enrichment analysis of the differential metabolites demonstrated pronounced involvement in pathways related to amino acid metabolism (such as, beta-alanine, cysteine, and methionine metabolism), energy metabolism (such as, citrate cycle and oxidative phosphorylation), and cofactor biosynthesis (such as, pantothenate and CoA and thiamine metabolism) (**Figure 6A**). Further analysis based on impact values and -In (P-value) highlighted thiamine metabolism, pantothenate and CoA biosynthesis, the tricarboxylic acid (TCA) cycle, alanine, aspartate, and glutamate metabolism, and cysteine and methionine metabolism (**Figure 6B**).

### 3.6 Network analysis

To enhance the comprehension of the interplay among DEPs, a PPI network was assembled using the STRING database (von Mering et al., 2005). The PPI network analysis revealed that DEPs formed interactive modules, particularly within pathways such as thermogenesis and diabetic cardiomyopathy (**Figure 6C**). A core interaction network comprising 40 proteins was identified, which suggested potential coordinated functions in the hypoxic adaptation.

Integrated proteomic and metabolomic data were used to map DEPs and differential metabolites onto shared metabolic pathways in Cytoscape software. In particular, proteins such as adenylate kinase 2 and Ndufs1 were linked to metabolites within pantothenic acid, thiamine, and S-adenosylhomocysteine. This highlights potential points of cross-talk between protein expression and metabolic flux (**Figure 6D**).

### 3.7 Validation of key protein expression via western blot

**Figure 7:**
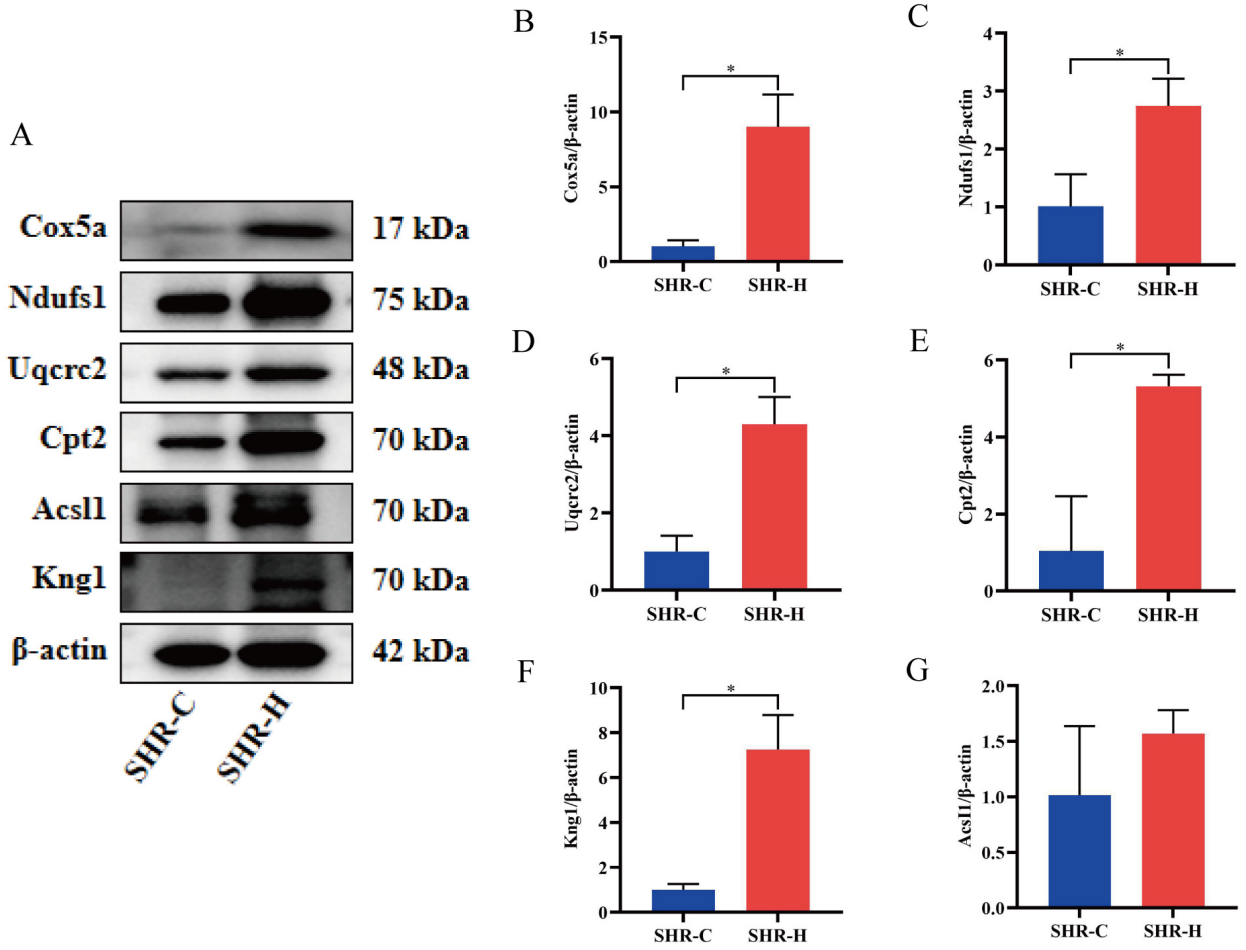
Changes in the protein expression levels of cytochrome c oxidase subunit 5A (Cox5a), NADH:ubiquinone oxidoreductase core subunit S1 (Ndufs1), ubiquinol-cytochrome c reductase core protein 2 (Uqcrc2), carnitine palmitoyltransferase (Cpt2), acyl-CoA synthetase long chain family member 1 (AcsI1), and kininogen 1 (Kng1) in the abdominal aorta of spontaneously hypertensive rats (SHR) after exposure to high-altitude hypoxic conditions. **(A)** Western blot images. Corresponding densitometric quantification. Band intensity was normalized to β−actin and quantified using Gel−Pro Analyzer 4 software. **(B)** Cox5a. **(C)** Ndufs1. **(D)** Uqcrc2. **(E)** Cpt2. **(F)** Kng1. **(G)** AcsI1. Data are shown as the mean ± SD (n = 3). SHR-C and SHR-H refer to the SHR control group and SHR high-altitude hypoxia group, respectively. *P < 0.05 vs. SHR-C (*t*-test).

The expression levels of several key DEPs were assessed to validate the proteomics results. Consistent with the TMT data, the Cox5a, Cpt2, Ndufs1, and Uqcrc2 protein levels were significantly upregulated in the SHR-H group, whereas Kng1 protein expression was significantly increased (P < 0.05) (**Figures 7A–F**). The expression of AcsI1 did not significantly differ between the two groups (P > 0.05) (**Figure 7G**).

## 4 Discussion

This study is the first to integrate quantitative TMT proteomic and untargeted metabolomic analyses in order to systematically elucidate the intricate high-altitude hypoxia-induced molecular remodeling of SHR aortic tissue. This was accompanied by an adaptive pattern characterized by a pronounced decrease in blood pressure. The key findings indicate that high-altitude hypoxia may exert antihypertensive effects by inducing vascular metabolic remodeling and functional adaptation via enhanced mitochondrial energy metabolism and the inhibition of inflammation and stress responses. Chronic hypoxic exposure does not universally confer protective effects. Previous studies have demonstrated it can increase oxidative stress, thereby promoting excessive proliferation and migration of pulmonary artery smooth muscle cells and fibroblasts, along with extracellular matrix deposition, ultimately leading to vascular remodeling and elevated vascular resistance (Shimoda, 2020). Moreover, hypoxic exposure alters the function and phenotype of endothelial cells, resulting in increased reactive oxygen species production, heightened inflammation, changes in vascular tone, and increased endothelial barrier permeability (Tóth and Jeney, 2025). Therefore, our findings should be interpreted within in the specific context of SHR exposed to hypoxia at an altitude of 4,300 m for 10 weeks, caution is warranted when extrapolating these results to other hypoxic conditions or disease states.

The SHR are inbred strain and the most studied model of essential hypertension in humans. The SHR strain was developed in Kyoto in the 1960s through selective inbreeding of Wistar rats for hypertension (Okamoto and Aoki, 1963). Blood pressure in SHRs begins to increase at 5–6 weeks of age and is fully established at 15–20 weeks of age. The characteristics of SHRs include phenotypic abnormalities, such as spontaneous hypertension, cardiac hypertrophy, and vascular dysfunction (Doggrell and Brown, 1998). Chronic high-altitude hypoxic exposure requires subjecting rats to a high-altitude hypoxic environment for 3–6 weeks (McMorrow et al., 2011). Considering these factors, SHRs were exposed to high-altitude hypoxia for 10 weeks to establish an SHR model of chronic high-altitude hypoxia exposure.

Blood pressure is determined using cardiac output and peripheral vascular resistance. Cardiac output depends on the volume of blood filling the heart and HR; the volume of blood filling the heart is influenced by the diastolic and systolic capabilities of the myocardium. Peripheral vascular resistance can be determined using the diameter and tension of blood vessels, particularly the arterioles and small arteries. The anatomical structure of blood vessels, functional state of the vascular smooth muscle, and function of endothelial cells affect vascular resistance (de Carvalho Ribeiro et al., 2023). Therefore, the abdominal aortas of the SHRs were analyzed. This artery is crucial for regulating blood pressure, particularly peripheral vascular resistance. The blood pressure values revealed that exposure to high-altitude hypoxia decreased SBP, DBP, and MBP in SHRs, consistent with a previous study reporting that high-altitude hypoxia could decrease SBP in SHRs through regulation of thyroid hormone levels and metabolic changes (Okamoto and Aoki, 1963).

TMT-tagged proteomic and untargeted metabolomic analyses were performed to map the proteins and metabolites in SHRs exposed to high-altitude hypoxia. Proteomics analysis revealed pronounced upregulation of a series of proteins closely associated with the mitochondrial electron transport chain, such as Cox5a (complex IV), Ndufs1 (complex I), and Uqcrc2 (complex III). This was accompanied by enrichment of the oxidative phosphorylation pathway in the KEGG analysis. Oxidative phosphorylation is a crucial process that generates ATP through the oxidation of glucose and other organic substances, which is crucial for cellular function (Fernández-Silva et al., 2003). This process is vital in cardiovascular disease and hypertension studies. Hypertension inhibits oxidative phosphorylation, decreases mitochondrial respiration and ATP production, and increases the generation of reactive oxygen species, thereby causing oxidative stress in cells and tissues (Gao and Shen, 2023). The findings of study indicates that, under high-altitude hypoxic conditions, the efficiency of mitochondrial ATP synthesis in vascular smooth muscle cells is enhanced. Correspondingly, the key rate-limiting enzyme Cpt2, which facilitates fatty acid entry into mitochondria for β-oxidation, was markedly upregulated at the protein level; metabolomic data revealed a substantial accumulation of various long-chain and medium-chain acylcarnitines, such as oxopalmitoleylcarnitine. Acylcarnitines are intermediate products of fatty acid β-oxidation, and their accumulation typically indicates increased flux through this metabolic pathway. This metabolic substrate shift from glucose oxidation to fatty acid oxidation may supply the vasculature with fuel for increased energy output under hypoxic conditions and may indirectly regulate vascular tone and remodeling by modulating the activity of intracellular energy sensors, calcium homeostasis, and reactive oxygen species production (Kalucka et al., 2018).

In contrast to the enhanced energy metabolism, this study revealed a broad suppression of inflammation and stress responses. Proteomics analysis identified notable downregulation of multiple relevant proteins, and GO enrichment confirmed their involvement in processes such as responses to stimuli. This inhibition may directly alleviate inflammation and oxidative stress in the vascular walls. A notable discrepancy was observed for Kng1, which was confirmed as downregulated via TMT proteomics but confirmed as upregulated via western blotting. This observed discrepancy is likely due to methodological differences between TMT-based proteomics and antibody based western blotting, including the effects of post-translational modifications, such as Kng1 glycosylation, which may influence peptide ionization efficiency or antibody epitope accessibility. Variations in sample processing and the differential detection of Kng1 proteolytic fragments may also have contributed (Martins et al., 2026; Myers et al., 2019). Crucially, this inconsistency does not compromise the overall inhibitory trend, which is consistently validated by the upregulation of Cox5a, Cpt2, Ndufs1, and Uqcrc2 across both analytical platforms. The case of Kng1 exemplifies the complexity of high-altitude hypoxic adaptation and highlights the need for future cell-type-specific investigations using orthogonal approaches.

KEGG pathway enrichment analysis of DEPs indicated that high-altitude hypoxia primarily affected energy-related pathways, as well as cofactor metabolism pathways. This collectively reflects the remodeling of the metabolic network. To elucidate the intrinsic logic of this remodeling, a joint protein–metabolite joint network was constructed. The analysis revealed that key DEPs and differentially expressed metabolites markedly converged on the TCA cycle—a core energy metabolism pathway. This finding is consistent with the hypothesis that energy metabolism is enhanced in SHRs under high-altitude hypoxia, but causality cannot be established form correlative data alone. Hence, changes in upstream protein expression remodel downstream metabolites. In addition, this finding provides a logically closed loop linking enhanced fatty acid oxidation to activated mitochondrial oxidative phosphorylation. The observed accumulation of acylcarnitines and changes in TCA cycle intermediates represent two consecutive steps in which wherein fatty acid catabolism end-products enter and drive the mitochondrial energy generation hub. Therefore, network analysis demonstrated that high-altitude hypoxia-induced changes were not isolated molecular events, but rather a coordinated metabolic program remodeling centered around the TCA cycle as the key integrative node. This strongly supports the notion that enhanced energy metabolism is the cornerstone of the entire adaptation program, and the energy produced alongside metabolic signals may provide the material and regulatory basis for other adaptive changes, such as the suppression of inflammatory responses (Liu et al., 2023b; Martínez-Reyes and Chandel, 2020; Venediktova et al., 2013). Accordingly, the integrative multi-omics network analysis corroborates proteome-driven metabolic reprogramming at the systemic level and reveals the central role of the TCA cycle.

This study has several limitations: First, only representative upregulated proteins were verified by the western blot, whereas downregulated proteins were not included, making the validation incomplete. Second, a lack of immunofluorescence/immunohistochemistry spatial localization data prevents differentiation of specific responses in various vascular cell types, and whole tissue homogenate analysis may obscure key cellular-level changes. Third, the inconsistent expression patterns of Kng1 observed in proteomics and western blot assays remain to be clarified, and further require cross-validation with multiple methods and antibodies is required. Finally, no experiments were conducted, thus the proposed mechanism of “enhanced energy metabolism mediating blood pressure reduction” remains a correlative inference, necessitating subsequent targeted intervention studies for confirmation. Therefore, future research will increase the sample size, supplement validation of downregulated proteins and spatial localization experiments, and establish causal relationships through pharmacological or genetic interventions to strengthen the reliability of the conclusions.

In summary, by integrating multi-omics technologies, this study provides correlative evidence for the molecular basis of high-altitude hypoxia-induced blood pressure reduction in SHRs, which warrants further functional validation. The core mechanism may be associated with activated mitochondrial oxidative phosphorylation and fatty acid oxidation to ensure ATP supply and a potentially broad inhibition of inflammation and stress responses to conserve energy and alleviate vascular injury. This study provides new mechanistic insights into the cardiovascular effects of high-altitude hypoxic environments and offers potential novel strategies for metabolic intervention in hypertension.

## Data Availability

The datasets presented in this study can be found in online repositories. The names of the repository/repositories and accession number(s) can be found in the article/**Supplementary Material**.
