# Supplementary material for: Integrated proteomics and metabolomics reveal mechanisms of blood pressure reduction in spontaneously hypertensive rats under hypoxic conditions
Source: Front Physiol. 2026 Jul 20;17:1859072. doi: 10.3389/fphys.2026.1859072 (PMC13429394; doi:10.3389/fphys.2026.1859072)
Supplement: Supplementary file 1 [file DataSheet1.pdf]

Tables

Table 1 Differentially expressed proteins in the abdominal aorta tissue samples of SHR-C and SR-H rats exposed to a high-altitude hypoxic environment

Number	Gene name	Accession	Mass	Protein names	P-value	Fold change	Regulation
1	Ucp1	A6IYF5	33212	Mitochondrial brown fat uncoupling	0.020	8.112	Up
2	RGD1311037	A6HQH5	32628	cardiolipin synthase	0.005	4.952	Up
3	Gpd1	A6KCH1	37453	Glycerol-3-phosphate dehydrogenase	0.009	4.768	Up
4	LOC100360357	A0A8I6ADD5	7425	Cytochrome c oxidase polypeptide	0.034	4.692	Up
5	Cpt2	P18886	74110	Carnitine O-palmitoyltransferase II	0.009	4.583	Up
6	COX2	A0A097PE04	25894	Cytochrome c oxidase subunit 2	0.017	4.510	Up
7	Fasn	A0A8I6A2Z2	274557	Fatty acid synthase	0.003	4.406	Up
8	Pdhb	A0A0G2KAM3	45115	Pyruvate dehydrogenase E1	0.011	4.389	Up
9	Acaa2	A6KRG3	41885	Acetyl-Coenzyme A acyltransferase 2	0.011	4.259	Up
10	Cox5a	A0A8L2QDJ0	15312	Cytochrome c oxidase polypeptide	0.018	4.241	Up
11	Etfa	A0A8L2QBI2	34707	Electron transfer flavoprotein subunit	0.017	4.171	Up
12	Acadm	A0A8I5Y8D9	45568	Medium-chain specific acyl-CoA	0.013	4.102	Up
13	rCG_29836	A6IMZ9	10405	RCG29836	0.005	4.061	Up
14	Decr1	A6IIA8	36133	2,4-dienoyl-CoA reductase	0.009	4.042	Up
15	Acadvl	F7FEX1	70821	Very long-chain specific acyl-CoA	0.010	3.929	Up
16	Idh3g	A0A0G2K4Q0	42380	Isocitrate dehydrogenase [NAD]	0.015	3.872	Up
17	Sdhc	A0A8I5Y7P2	16999	Succinate dehydrogenase cytochrome	0.007	3.800	Up
18	Acadl	A6KFD6	47873	Long-chain specific acyl-CoA	0.011	3.747	Up
19	Uqcr10	A0A9K3Y7E2	7462	Complex III subunit 9	0.003	3.675	Up
20	Acad10	A0A8I6A4Z6	61929	Aldehyde dehydrogenase 2 family	0.012	3.665	Up
21	Hadhb	A6HAE3	51414	Trifunctional enzyme subunit beta	0.008	3.614	Up
22	Cabc1	A6JGG2	72226	Atypical kinase COQ8A	0.011	3.593	Up
23	Sucla2	B2RZ24	47388	Succinate-CoA ligase subunit beta	0.015	3.583	Up
24	Etfb	A6JAH9	27687	Electron transfer flavoprotein subunit	0.012	3.527	Up
25	Idh3a	A6J4L7	41177	Isocitrate dehydrogenase [NAD]	0.016	3.485	Up
26	Ndufa4	A6IDY9	9327	Ndufa4, mitochondrial complex	0.003	3.461	Up
27	rCG_52466	A6K0M2	11605	RCG52466	0.017	3.431	Up
28	Echdc1	A0A8I6AT16	50698	Ethylmalonyl-CoA decarboxylase	0.010	3.426	Up
29	Gpd1l1	D3ZAP9	38066	Glycerol-3-phosphate dehydrogenase	0.010	3.418	Up
30	Cox4i1	A0A8I5ZUV1	25275	Cytochrome c oxidase subunit 4	0.012	3.408	Up

31	Dlst	A6JE02	48899	Dihydrolipoyllysine-residue	0.011	3.397	Up
32	Them2_predicted	A6KLF5	15270	Thioesterase superfamily member 2	0.027	3.377	Up
33	LOC102555814	A0A8I5XZX9	189760	Uncharacterized LOC102555814	0.015	3.370	Up
34	Ogdh	A0A8L2R156	118026	2-oxoglutarate dehydrogenase	0.020	3.344	Up
35	rCG_61339	A6HAE4	82665	enoyl-CoA hydratase	0.012	3.313	Up
36	Qpc	A6HEC7	9849	Cytochrome b-c1 complex subunit 8	0.011	3.252	Up
37		A0A096XNM4	43042	Cytochrome b	0.011	3.240	Up
38	Cox6c	A6HR20	8455	Cytochrome c oxidase subunit 6C	0.006	3.220	Up
39	Sdhd	A0A8I5ZSF4	16415	Succinate dehydrogenase	0.009	3.200	Up
40	Aco2	A0A8I5ZLT6	85141	Aconitate hydratase, mitochondrial	0.006	3.173	Up
41	Uqcrc1	A6I395	52849	Ubiquinol-cytochrome c reductase	0.031	3.153	Up
42	Acsf2	A0A8I6GEJ7	65838	Medium-chain acyl-CoA ligase	0.005	3.148	Up
43	Ndufv2	A0A8I5ZXA6	27859	NADH dehydrogenase [ubiquinone]	0.008	3.137	Up
44	Dld	A0A8I5ZXS2	53267	Dihydrolipoyl dehydrogenase	0.011	3.131	Up
45	Me1	A0A0G2K1S6	60915	Malic enzyme	0.001	3.116	Up
46	Ndufa5	A6IE84	13412	NADH dehydrogenase [ubiquinone]	0.010	3.086	Up
47	Hadhsc	A6HVS6	34448	3-hydroxyacyl-CoA dehydrogenase	0.000	3.055	Up
48	Etfdh	A0A8I5Y801	64980	Electron transfer flavoprotein-	0.024	3.020	Up
49	Cpt1b	A6K7M6	88217	Carnitine O-palmitoyltransferase 1	0.014	3.006	Up
50	rCG_50860	A6KCJ7	41967	RCG50860, isoform CRA_b	0.011	2.976	Up
51	Tkt	P50137	67644	Transketolase (TK)	0.010	2.965	Up
52	Slc25a20	A6I375	33071	Solute carrier family 25	0.015	2.937	Up
53	rCG_20813	A6JEJ6	6998	NADH dehydrogenase [ubiquinone]	0.008	2.918	Up
54	Mdh2	A0A8I6ABC9	34569	Malate dehydrogenase	0.014	2.910	Up
55	Acsl1	A0A8L2R6L8	78252	Long-chain-fatty-acid--CoA ligase	0.016	2.904	Up
56	Cox6b1	A0A8I6A8X0	10071	Cytochrome c oxidase subunit	0.004	2.900	Up
57	rCG_38845	A6KAA1	16777	NADH dehydrogenase [ubiquinone]	0.011	2.893	Up
58	Dlat	A6J4E7	67166	Acetyltransferase component of	0.014	2.877	Up
59	Mpc1	P63031	12455	Mitochondrial pyruvate carrier 1	0.004	2.857	Up
60	Ndufb6	A0A8I6GCX1	11857	NADH dehydrogenase [ubiquinone]	0.011	2.856	Up
61	ND1	D2E6L7	36062	NADH-ubiquinone oxidoreductase	0.003	2.820	Up
62	Ndufab1_predicted	A6I8V9	17514	Acyl carrier protein	0.029	2.786	Up
63	Uqcrc2	A0A8I6A888	48439	Ubiquinol cytochrome c reductase	0.005	2.771	Up
64	Acly	A6HJ39	120781	ATP-citrate synthase	0.000	2.747	Up
65	Cs	A0A8I6GES0	55579	Citrate synthase	0.004	2.734	Up

66	RGD1563422_predicted	A6IDH0	14258	Mitochondrial pyruvate carrier	0.003	2.720	Up
67	Ndufa7	A9UMV9	12500	NADH dehydrogenase [ubiquinone]	0.007	2.710	Up
68	Ndufs3_predicted	A6HN86	34413	NADH dehydrogenase [ubiquinone]	0.009	2.706	Up
69	Timm44	A0A8I5ZV20	54507	Mitochondrial import inner	0.012	2.701	Up
70	Fh	P14408	54465	Fumarate hydratase	0.008	2.688	Up
71	Gpd2	A0A0G2K1F9	78099	Glycerol-3-phosphate dehydrogenase	0.006	2.687	Up
72	Akr1c15	A0A387KC71	37195	Aldo-keto reductase family 1,	0.012	2.682	Up
73	rCG_32910	A6HD70	17918	RCG32910, isoform CRA_b	0.002	2.678	Up
74	Clybl	A0A8I6B1P6	37608	Citramalyl-CoA lyase	0.024	2.625	Up
75	Ndufa6_predicted	A6HT69	15224	NADH dehydrogenase [ubiquinone]	0.013	2.618	Up
76	Echs1	A6HXG6	31516	Enoyl-CoA hydratase	0.019	2.600	Up
77	Ndufa2	A0A8I5ZKJ3	11017	NADH dehydrogenase [ubiquinone]	0.012	2.595	Up
78	Cox8h	A6HXL6	7549	Cytochrome c oxidase subunit 8	0.009	2.584	Up
79	Ndufs2	Q641Y2	52562	NADH dehydrogenase [ubiquinone]	0.009	2.577	Up
80	rCG_55630	A6JQU0	40493	NADH dehydrogenase [ubiquinone]	0.009	2.555	Up
81	Thrsp	A6I696	17085	Thyroid hormone responsive	0.003	2.555	Up
82	Suclg1	A0A0H2UHE1	37560	Succinate--CoA ligase [ADP/GDP-	0.005	2.542	Up
83	Pc	A0A0G2JTL5	140005	pyruvate carboxylase	0.007	2.541	Up
84	Ndufs1	A0A8I5ZXM3	78939	NADH-ubiquinone oxidoreductase 75	0.014	2.540	Up
85	Ppif	A0A8L2Q6S2	21211	Peptidyl-prolyl cis-trans isomerase	0.013	2.531	Up
86	Pck1	A6KKZ5	69416	Phosphoenolpyruvate carboxykinase	0.035	2.523	Up
87	Mecr	A0A140TAE6	40335	Enoyl-[acyl-carrier-protein] reductase	0.007	2.518	Up
88	Impa2	A0A0G2JUN5	31221	Inositol-1-monophosphatase	0.012	2.502	Up
89	rCG_35301	A6HIA7	29820	Prohibitin	0.015	2.481	Up
90	Fahd1	A6HCX6	24480	Acylpyruvase FAHD1	0.033	2.479	Up
91	ADSSL1	A6KBV5	50250	Adenylosuccinate synthetase isozyme	0.025	2.456	Up
92	Acat1	A6J4J7	44695	Acetyl-CoA acetyltransferase	0.000	2.455	Up
93	Sdhaf2	A0A8I5ZL70	15766	Succinate dehydrogenase complex	0.008	2.454	Up
94	Ndufb8	A6JHG3	21959	NADH dehydrogenase [ubiquinone]	0.005	2.451	Up
95	Ndufv1	A0A8I6ALF5	50731	NADH dehydrogenase [ubiquinone]	0.011	2.451	Up
96	Tufm_predicted	A6I973	49522	Elongation factor Tu	0.008	2.436	Up
97	Sdhb_predicted	A6ITQ1	33324	Succinate dehydrogenase	0.030	2.413	Up
98	Uqcrfs1	A6J7M2	29446	Cytochrome b-c1 complex subunit	0.014	2.404	Up
99	Cox7b	A0A8I5ZZJ9	11825	Cytochrome c oxidase subunit 7B	0.016	2.387	Up
100	Nudt13	B2GV51	39334	NAD(+) diphosphatase	0.016	2.379	Up

101	Cox7a2	A0A8L2QTB7	9296	Cytochrome c oxidase subunit 7A2	0.008	2.375	Up
102	Gys2	A6IMU2	77548	Glycogen [starch] synthase	0.003	2.374	Up
103	Cox5b	A6INE7	13915	Cytochrome c oxidase subunit 5B	0.014	2.371	Up
104	Tymp	A0A8I5ZWG1	29285	Thymidine phosphorylase	0.009	2.367	Up
105	Prxl2a	A0A8I6G216	26535	Peroxiredoxin-like 2A	0.004	2.333	Up
106	Lrpprc	A6H9I4	156680	Leucine-rich PPR-motif containing,	0.011	2.331	Up
107	Idh3b	A0A8L2Q4B4	51050	Isocitrate dehydrogenase [NAD]	0.006	2.328	Up
108	Ndufc2	A6I691	14359	NADH dehydrogenase [ubiquinone]	0.008	2.325	Up
109	Rmdn1	A0A0G2K167	36795	Regulator of microtubule dynamics	0.021	2.320	Up
110	Acot2	A0A8I6A1M5	49892	Acyl-CoA thioesterase 2	0.004	2.314	Up
111	rCG_32945	A6HCX0	20859	NADH dehydrogenase [ubiquinone]	0.032	2.312	Up
112	Ndufb3	A0A8I5Y9A4	12268	NADH dehydrogenase [ubiquinone]	0.013	2.287	Up
113	Letm1	A0A8I5ZLQ4	85774	Mitochondrial proton/calcium	0.016	2.266	Up
114	ND2	Q06QE9	38598	NADH-ubiquinone oxidoreductase	0.017	2.264	Up
115	Mrpl12	A6HLD8	21713	Ribosomal protein, mitochondrial,	0.008	2.260	Up
116	rCG_41639	A6IH46	10895	Fatty acid-binding protein	0.008	2.252	Up
117	Ndufb5_predicted	A6IHQ7	21664	NADH dehydrogenase [ubiquinone]	0.010	2.251	Up
118	Ndufa9	A0A8I6A3W0	50062	NADH dehydrogenase [ubiquinone]	0.006	2.249	Up
119	Ucp3	A6I6N5	34015	Putative mitochondrial transporter	0.003	2.239	Up
120	Fasn	A6HLK4	146482	Fatty acid synthase	0.001	2.236	Up
121	Cox7a2l	A0A8I6GHX2	14978	Cytochrome c oxidase subunit 7A2	0.011	2.229	Up
122	Ndufb11_predicted	A6JZT7	17634	NADH dehydrogenase [ubiquinone]	0.010	2.229	Up
123	Glul	A0A8I5Y1V3	43903	Glutamine synthetase	0.024	2.226	Up
124	Ndufv3	A0A8I6AEG4	48622	NADH:ubiquinone oxidoreductase	0.016	2.225	Up
125	Mtfp1	A6IKE5	18366	Mitochondrial fission process protein	0.007	2.223	Up
126	Ptges2_predicted	A6JU62	43452	Prostaglandin E synthase 2	0.006	2.221	Up
127	Coq7	A6I8H3	23870	5-demethoxyubiquinone hydroxylase	0.010	2.218	Up
128	Oxnad1	A0A0G2K466	34847	Oxidoreductase NAD-binding	0.026	2.192	Up
129	Hsdl2	A0A8L2QRI7	58459	Hydroxysteroid dehydrogenase-like	0.007	2.190	Up
130	rCG_44686	A6I5A6	11413	RCG44686, isoform CRA_a	0.007	2.179	Up
131	Ndufs4	A0A8I5ZK55	23473	NADH dehydrogenase [ubiquinone]	0.004	2.174	Up
132	Scd3	A0A8I5ZQY8	41174	Similar to stearoyl-coenzyme A	0.002	2.172	Up
133	rCG_20695	A6JEA9	12576	RCG20695, isoform CRA_b	0.007	2.171	Up
134	Auh	A0A8I5Y7Q4	31664	AU RNA binding methylglutaconyl-	0.008	2.154	Up
135	Pygl	A6HBY8	97483	Alpha-1,4 glucan phosphorylase	0.005	2.154	Up

136	Bckdha	A0A8I6GGA5	50202	2-oxoisovalerate dehydrogenase	0.007	2.137	Up
137	Cyc1_predicted	A6HS83	35435	Cytochrome c-1 (Predicted), isoform	0.013	2.132	Up
138	Slc2a4	A0A8I6B1R2	53212	Solute carrier family 2, facilitated	0.005	2.128	Up
139	Dhrs9	Q8VD48	35214	Dehydrogenase/reductase SDR	0.002	2.126	Up
140	Ak2	A0A0G2JSG6	25529	Adenylate kinase 2	0.015	2.126	Up
141	Atad3a	A6IUR9	66759	ATPase family, AAA domain	0.013	2.124	Up
142	Mrps35_predicted	A6IN39	36207	Mitochondrial ribosomal protein S35	0.011	2.122	Up
143	Ndufs8	A0A8I6AL00	26878	NADH dehydrogenase [ubiquinone]	0.017	2.121	Up
144	Prdx3	A0A8I5ZMG4	28299	Thioredoxin-dependent peroxide	0.019	2.121	Up
145	Ndufs7	A0A8I6AES7	24047	NADH dehydrogenase [ubiquinone]	0.000	2.117	Up
146	Ndufa12_predicted	A6IG10	17178	NADH dehydrogenase [ubiquinone]	0.000	2.114	Up
147	D2hgdh	A0A8L2R544	60842	D-2-hydroxyglutarate dehydrogenase	0.006	2.109	Up
148	ND5	A0A096XKT9	68584	NADH-ubiquinone oxidoreductase	0.013	2.081	Up
149	Uqcrb	B2RYS2	13559	Cytochrome b-c1 complex subunit 7	0.007	2.061	Up
150	Plin	A6JC64	55862	Perilipin	0.023	2.051	Up
151	Taco1	B2RYT9	32754	Translational activator of cytochrome	0.011	2.047	Up
152	Phb2	Q5XIH7	33312	Prohibitin-2	0.018	2.046	Up
153	RGD1310475_predicted	A6JHA2	34465	4-hydroxy-2-oxoglutarate aldolase	0.028	2.043	Up
154		Q71DI1	11284	Dermcidin	0.004	2.037	Up
155	Coa6	A0A8I5ZL79	9339	Cytochrome c oxidase assembly	0.012	2.035	Up
156	Mrps28	A0A0G2K8L9	24300	Mitochondrial ribosomal protein S28	0.005	2.026	Up
157	Gcdh	A0A8I5ZN89	48627	Glutaryl-CoA dehydrogenase	0.023	2.021	Up
158	Hmgcl	A0A8I5ZY90	34879	Hydroxymethylglutaryl-CoA lyase	0.012	2.021	Up
159	Zadh2_predicted	A6K5K8	40476	Zinc binding alcohol dehydrogenase,	0.008	2.010	Up
160	rCG_58764	A6JLI5	14342	Small ribosomal subunit protein	0.006	2.009	Up
161	Suox	A0A8I6ABF6	61117	sulfite oxidase	0.009	2.004	Up
162	Kng1	A0A8L2R8P7	48444	Kininogen 1	0.001	0.245	Down
163	Pla2g2a	A6ITI9	16294	Phospholipase A2	0.035	0.298	Down
164	Crip1	A0A8I6A364	13513	Cysteine rich protein 1	0.001	0.337	Down
165	Mfge8	A6JC45	47413	Milk fat globule-EGF factor 8 protein	0.010	0.352	Down
166	Hp	A0A0H2UHM3	38389	Haptoglobin	0.001	0.366	Down
167	rCG_22430	A6IPA6	63765	RCG22430	0.031	0.369	Down
168	Mustn1	A0A8I6AU05	10569	Musculoskeletal embryonic nuclear	0.002	0.382	Down
169	Fhl5	A0A8I5ZQD5	36626	Four and a half LIM domains protein	0.004	0.407	Down
170	rCG_46036	A6ICF5	195109	RCG46036	0.001	0.409	Down

171	Akap9	A0A0G2K548	441620	A-kinase anchoring protein 9	0.007	0.420	Down
172	Sorbs2	A0A8I5ZQ23	72336	Sorbin and SH3 domain containing 2	0.002	0.422	Down
173	Lyz2	A0A077S1I6	16718	lysozyme (EC 3.2.1.17) (1,4-beta-N-	0.011	0.423	Down
174	Zmynd8	A0A8I5XZV4	139170	Zinc finger, MYND-type containing	0.002	0.433	Down
175	Fabp1	A6IA66	14273	Fatty acid-binding protein	0.035	0.435	Down
176	Grem2_predicted	A6JGB0	19348	Gremlin	0.013	0.453	Down
177	Folr2_predicted	A6I6W6	29169	Folate receptor 2 (Fetal) (Predicted),	0.018	0.458	Down
178	Ces1c	D3ZGK7	64219	Carboxylic ester hydrolase	0.008	0.461	Down
179	Palld	A0A8I5ZQM3	108341	Palladin-like 1	0.001	0.465	Down
180	Ccl9	A6HHI4	13101	C-C motif chemokine ligand 9	0.016	0.468	Down
181	Ass	A6JU24	46496	Argininosuccinate synthase	0.034	0.473	Down
182	Prg4	A0A8I6A1N7	128544	Proteoglycan 4	0.008	0.485	Down
183	rCG_52348	A6K0J1	26613	RCG52348	0.048	0.487	Down
184	Plpp1	A0A8I5ZMN4	31986	Phospholipid phosphatase 1	0.010	0.492	Down
185	rCG_61008	A6JJM1	11706	High mobility group protein HMG-	0.046	0.492	Down
