# Supplementary material for: Integrated proteomics and metabolomics reveal mechanisms of blood pressure reduction in spontaneously hypertensive rats under hypoxic conditions
Source: Front Physiol. 2026 Jul 20;17:1859072. doi: 10.3389/fphys.2026.1859072 (PMC13429394; doi:10.3389/fphys.2026.1859072)
Supplement: Supplementary file 2 [file DataSheet2.pdf]

Table 2 List of significantly regulated metabolites in the abdominal aorta tissue samples of SHR-H rats compared to SHR-C rats using UHPLC/MS

Number	Class	Metabolite name	Fold change	P value	Regulation
1	Fatty Acyls	Oxopalmitoleylcarnitine	4.589	0.000	Up
2	Prenol lipids	Curcumenol	3.574	0.000	Up
3		(2-aminoethoxy)[-2-[hexadec-9-enoyloxy]-3-(hexadecanoyloxy)propoxy]phosphinic acid	2.991	0.000	Up
4	Benzopyrans	HU-210	5.551	0.000	Up
5	Isocoumarins and derivatives	3-(2,4-Dihydroxypentyl)-6,8-dimethoxyisochromen-1-one	2.881	0.000	Up
6	Organonitrogen compounds	Chlorambucil	2.634	0.000	Up
7	Fatty Acyls	3-Hydroxyhexadecanoylcarnitine	3.956	0.001	Up
8	Fatty Acyls	4,5-Epoxy-7Z,10Z,13Z,16Z,19Z-docosapentaenoic acid, methyl ester	4.556	0.001	Up
9		Caryophyllene_epoxide	3.239	0.001	Up
10		Oxododecanoylcarnitine	4.093	0.001	Up
11	Small peptides	Homoserine	3.994	0.001	Up
12	Fatty Acyls	Oxopalmitoylcarnitine	5.681	0.001	Up
13	Fatty Acyls	cis-5-Tetradecenoylcarnitine	3.268	0.001	Up
14	Small peptides	Timonacic	33.712	0.002	Up
15	Organometalloid compounds	Cyclohexyl(phenyl)[3-(piperidin-1-yl)propyl]silanol	5.278	0.002	Up
16	Azolidines	5-Isopropyl-5-methylhydantoin	4.620	0.002	Up
17	Flavonoids	4-Methoxyphlorizin	9.944	0.002	Up
18	Fatty Acyls	Thromboxane B2	2.737	0.003	Up
19	Fatty Acids and Conjugates	Pantothenic acid	2.631	0.003	Up
20	Quinolines and derivatives	Bathophenanthroline	3.126	0.006	Up
21	Diazines	Thiamine	2.901	0.006	Up
22		trans-Hexadec-2-enoyl_carnitine	3.558	0.007	Up
23	Fatty Acyls	3-Hydroxyoleylcarnitine	5.516	0.007	Up
24	Fatty Acyls	Daminozide	3.932	0.007	Up
25	Steroids and steroid derivatives	Alfacalcidol	2.623	0.008	Up
26	Glycerophospholipids	1,2-Dipalmitoleoyl-sn-glycero-3-phosphoethanolamine	3.369	0.008	Up
27		2-Aminomuconate semialdehyde	3.076	0.008	Up
28	Glycerophospholipids	LysoPE(16:1(9Z)/0:0)	2.836	0.009	Up
29	Glycerophospholipids	3,5,9-Trioxa-4-phosphatetracosan-1-aminium, 7-(acetyloxy)-24-carboxy-4-hydroxy-N,N,N-trimethyl-, inner salt, 4-oxide, (R)-	4.354	0.010	Up
30	Fatty Acyls	Linoleoylcarnitine	3.847	0.010	Up
31	Polycyclic aromatic polyketides	Damnacanthal	3.266	0.013	Up
32	Steroids and steroid derivatives	(22E,24R)-Stigmasta-4,22-diene-3,6-dione	5.092	0.015	Up
33	Quinolines and derivatives	3-Quinolinecarboxamide, 1,4-dihydro-6-(1-methylethyl)-4-oxo-1-pentyl-N-tricyclo[3.3.1.1(3,7)]dec-1-yl-	3.241	0.015	Up
34	Fatty Acyls	2-Hydroxyoctanoic acid	3.762	0.016	Up
35	Fatty Acids and Conjugates	3-Hydroxyoctanoic acid	3.762	0.016	Up

36	Fatty Acyls	5-Hydroxyvalproic acid	3.762	0.016	Up
37	Diazines	6-Amino-1-butyl-5-cyclopentylamino-1H-pyrimidine-2,4-dione	34.607	0.016	Up
38	Fatty Acids and Conjugates	Fumaric acid	2.356	0.019	Up
39	Fatty Acids and Conjugates	Eicosapentaenoic acid	5.207	0.020	Up
40	Glycerophospholipids	LPG(18:1(9Z))	2.815	0.020	Up
41	Fatty Acyls	N-Palmitoyltaurine	3.511	0.020	Up
42	Ornithine alkaloids	Nicotine	6.660	0.021	Up
43	Lysine alkaloids	Anabasine	6.660	0.021	Up
44	Fatty Acyls	N-[(1R)-2-Hydroxy-1-methylethyl-9Z-octadecenamide	5.211	0.022	Up
45	Fatty Acids and Conjugates	Hydroxyisocaproic acid	5.032	0.023	Up
46	Fatty Acyls	5-Hydroxyhexanoic acid	5.032	0.023	Up
47	Fatty Acyls	2-Ethyl-2-hydroxybutyric acid	5.032	0.023	Up
48	Benzopyrans	7-Hydroxy-3-phenoxy-2-(trifluoromethyl)-4H-chromen-4-one	2.906	0.024	Up
49		Dipyridamole_(Persantine)	12.478	0.026	Up
50	Organooxygen compounds	Lotaustralin	4.259	0.027	Up
51	Steroids	Cholic acid	3.682	0.028	Up
52	Steroids and steroid derivatives	Ursocholic acid	3.682	0.028	Up
53	Steroids	Allocholic acid	3.682	0.028	Up
54	Steroids and steroid derivatives	omega-Muricholic acid	3.682	0.028	Up
55	Steroids and steroid derivatives	Hyocholic acid	3.682	0.028	Up
56	Steroids and steroid derivatives	alpha-Muricholic acid	3.682	0.028	Up
57	Steroids and steroid derivatives	beta-Muricholic acid	3.682	0.028	Up
58	Fatty Acyls	2,2-Dimethylsuccinic acid	4.932	0.032	Up
59		12-oxo-ETE	2.764	0.032	Up
60	Carboxylic acids and derivatives	Phe-Met-Arg-Phe-amide	2.998	0.033	Up
61	Quinolines and derivatives	8-Hydroxyquinoline-2-carboxamide	26.213	0.035	Up
62	Fatty Acids and Conjugates	3-Hydroxyvaleric acid	23.224	0.045	Up
63	Fatty Acyls	2-Methyl-3-hydroxybutyric acid	23.224	0.045	Up
64	Pyrrolopyridines	6-Methyl-1H,6H,7H-pyrrolo[2,3-c]pyridin-7-one	4.298	0.045	Up
65	Steroids and steroid derivatives	Drostanolone propionate	2.734	0.049	Up
66	Benzazepines	Varenicline	0.204	0.000	Down
67		Mizolastine (Mizollen)	0.361	0.001	Down
68	Naphthalenes	2-Chloro-5,6-dimethoxy-3-(2-naphthylthio)-p-benzoquinone	0.366	0.001	Down
69	Nucleosides	S-Adenosylhomocysteine	0.293	0.001	Down
70	Coumarins and derivatives	Coumarin-suberoylanilide hydroxamic acid	0.389	0.001	Down
71	Benzene and substituted derivatives	(2-Biphenyl)dicyclohexylphosphine	0.354	0.002	Down
72	Organonitrogen compounds	Lauramine oxide	0.195	0.003	Down
73	Carboxylic acids and derivatives	[(4,6-Dimethyl-2-pyrimidinyl)amino]acetic acid	0.225	0.004	Down
74	Small peptides	Carnosine	0.166	0.006	Down
75		Glucoconvallasaponin_B	0.277	0.011	Down
76	Organonitrogen compounds	Daltogen	0.199	0.013	Down
77	Cinnamic acids and derivatives	1,6-Bis-O-(4-hydroxycinnamoyl)glucose	0.335	0.018	Down

78	Small peptides	4-Hydroxybenzyl cyanide	0.005	0.018	Down
79	Piperidines	3-(3-Fluorobenzyl)piperidine	0.003	0.028	Down
80	Benzoxazoles	2-Methylbenzoxazole	0.166	0.038	Down
81	Indoles and derivatives	5-Hydroxyindole	0.166	0.038	Down
82	Organooxygen compounds	Tripropylene glycol	0.658	0.041	Down
